# Impact of Point-of-Care Rapid Diagnostic Tests on Antibiotic Prescription Among Patients Aged <18 Years in Primary Healthcare Settings in 2 Peri-Urban Districts in Ghana: Randomized Controlled Trial Results

**DOI:** 10.1093/cid/ciad328

**Published:** 2023-07-25

**Authors:** Alexander Adjei, Vida Kukula, Clement Tetteh Narh, Selase Odopey, Emmanuel Arthur, Gabriel Odonkor, Michael Matey Mensah, Piero Olliaro, Philip Horgan, Sabine Dittrich, Catrin E Moore, Olawale Salami, Elizabeth Awini, Juvenal Nkeramahame, John Williams, Rita Baiden

**Affiliations:** Department of Epidemiology, Dodowa Health Research Centre, Dodowa, Ghana; Department of Epidemiology, Dodowa Health Research Centre, Dodowa, Ghana; Department of Epidemiology, Dodowa Health Research Centre, Dodowa, Ghana; Department of Epidemiology and Biostatistics, Fred N. Binka School of Public Health, University of Health and Allied Sciences, Ho, Ghana; Department of Epidemiology, Dodowa Health Research Centre, Dodowa, Ghana; Department of Epidemiology, Dodowa Health Research Centre, Dodowa, Ghana; Department of Epidemiology, Dodowa Health Research Centre, Dodowa, Ghana; Department of Epidemiology, Dodowa Health Research Centre, Dodowa, Ghana; Department of Medical Affairs, FIND, Geneva, Switzerland; International Severe Acute Respiratory and Emerging Infection Consortium, Pandemic Sciences Institute, University of Oxford, Oxford, United Kingdom; Department of Medical Affairs, FIND, Geneva, Switzerland; Nuffield Department of Medicine, Big Data Institute, University of Oxford, Oxford, United Kingdom; Evidence & Impact Oxford, Oxford, United Kingdom; Department of Medical Affairs, FIND, Geneva, Switzerland; Nuffield Department of Medicine, Centre for Tropical Medicine and Global Health, University of Oxford, Oxford, United Kingdom; Deggendorf Institute of Technology, European Campus Rottal Inn, Pfarrkirchen, Germany; Nuffield Department of Medicine, Big Data Institute, University of Oxford, Oxford, United Kingdom; Centre for Neonatal and Paediatric Infection, Institute for Infection and Immunity, St. George's University of London, London, United Kingdom; Department of Medical Affairs, FIND, Geneva, Switzerland; Department of Epidemiology, Dodowa Health Research Centre, Dodowa, Ghana; Department of Medical Affairs, FIND, Geneva, Switzerland; Department of Epidemiology, Dodowa Health Research Centre, Dodowa, Ghana; Department of Epidemiology, Dodowa Health Research Centre, Dodowa, Ghana

**Keywords:** point-of-care test, antibiotics, antimicrobial resistance, adherence

## Abstract

**Background:**

Inappropriate antibiotic prescriptions are a known driver of antimicrobial resistance in settings with limited diagnostic capacity. This study aimed to assess the impact of diagnostic algorithms incorporating rapid diagnostic tests on clinical outcomes and antibiotic prescriptions compared with standard-of-care practices, of acute febrile illness cases at outpatient clinics in Shai-Osudoku and Prampram districts in Ghana.

**Methods:**

This was an open-label, centrally randomized controlled trial in 4 health facilities. Participants aged 6 months to <18 years of both sexes with acute febrile illness were randomized to receive a package of interventions to guide antibiotic prescriptions or standard care. Clinical outcomes were assessed on day 7.

**Results:**

In total, 1512 patients were randomized to either the intervention (n = 761) or control (n = 751) group. Majority were children aged <5 years (1154 of 1512, 76.3%) and male (809 of 1512, 53.5%). There was 11% relative risk reduction of antibiotic prescription in intervention group (RR, 0.89; 95% CI, .79 to 1.01); 14% in children aged <5 years (RR, 0.86; 95% CI, .75 to .98), 15% in nonmalaria patients (RR, 0.85; 95% CI, .75 to .96), and 16% in patients with respiratory symptoms (RR, 0.84; 95% CI, .73 to .96). Almost all participants had favorable outcomes (759 of 761, 99.7% vs 747 of 751, 99.4%).

**Conclusions:**

In low- and middle-income countries, the combination of point-of-care diagnostics, diagnostic algorithms, and communication training can be used at the primary healthcare level to reduce antibiotic prescriptions among children with acute febrile illness, patients with nonmalarial fevers, and respiratory symptoms.

**Clinical Trials Registration:**

NCT04081051.

## Teaser

Here, we report the main findings of the AMR Diagnostics Use Accelerator trial conducted in Ghana to evaluate the impact of a package of interventions on antibiotic prescribing and clinical outcomes for child and adult outpatients with acute febrile illness.

The new paradigm of healthcare focuses on adapting treatment preferences to patient-specific characteristics to optimize health outcomes; this includes antibiotic prescriptions for acute febrile illnesses [1]. At peripheral health centers with no diagnostic capacity, case management of febrile illnesses is challenging, and just-in-case use of antibiotics is prevalent. This routine practice of managing acute febrile illnesses is being challenged largely because of increasing antimicrobial resistance (AMR) fueled by the overprescription of inappropriate antibiotics as it is not always easy to diagnose the bacterial origin of an infectious disease [1].

Bacterial drug resistance is a major public health problem [2, 3]. Globally, bacterial AMR led to 1.27 million deaths and was associated with 4.95 million deaths in 2019 alone [4]. The proportion of antibiotic prescriptions in healthcare facilities in Ghana ranges from 33% to 55% in secondary and tertiary facilities and from 65% to 82% in primary facilities based on the level of care delivered [5–10]. Some laboratory-based nationwide surveillance studies in Ghana have reported multidrug resistance to commonly prescribed antibiotics [11, 12].

Inappropriate antibiotic prescribing is one of the causes of both AMR and inadequate management of acute febrile illnesses, both of which result in increased morbidity and mortality [13]. Nonadherence to prescriptions is another contributing factor to bacterial resistance, which is prevalent in the short-term treatment of acute infections [14]. Reasons for nonadherence include remission of symptoms, forgetfulness, poor attitude, and knowledge about antibiotic usage [15, 16]. Others include issues of adverse effects and problems with swallowing [17]. Inappropriate prescribing of antibiotics can adversely affect patient health and care with unfavorable outcomes [18]. Therefore, an adaptation in case management of infectious diseases at the primary healthcare level in low- and middle-income countries, including Ghana, that lack the diagnostic capacity to appropriately differentiate acute febrile illnesses and better target the use of antimicrobials is urgently needed.

New point-of-care diagnostic tools and therapeutic guidelines are urgently needed. Evidence shows that the use of point-of-care diagnostic testing (POCT) may help guide antibiotic prescription as it reduces the uncertainty in diagnosing and treating an infectious disease [19]. In the short term, POCTs are likely to reduce the number of inappropriate antibiotic prescriptions; in the medium and long term, POCTs are likely to help reduce bacterial resistance [20, 21]. For respiratory tract infections, POCTs have been used and their effectiveness has been assessed [22–24]. Although microbiological diagnostic tests are currently in use in hospitals, they are not usually available at the primary care level.

Our objective in this study was to compare the impact of a package of rapid diagnostic tests coupled with clinical algorithms and behavioral change communication for prescribers and caregivers on clinical outcomes and antibiotic prescriptions, with standard-of-care practices, in children and adolescents who present with acute febrile illnesses (defined as fever with no focus or respiratory tract infection that lasts for no longer than 7 days) at outpatient clinics in 2 districts in Ghana.

## METHODS

### Study Setting

The study was conducted in the Shai-Osudoku and Ningo-Prampram districts in the Greater Accra Region, Ghana, at 4 public health facilities with basic laboratory services, 2 in each district. The Shai-Osudoku District Hospital is a 140-bed public hospital, St. Andrew's Catholic Hospital and Prampram Polyclinic are 30-bed facilities, and Old Ningo is a 3-bed health center. Prampram Polyclinic and Old Ningo were used as satellite sites of Shai-Osudoku District Hospital. Patients at these facilities were attended to by either medical doctors or physician assistants who were university-trained for 4 years.

### Study Design

This was an open-label, prospective, comparative, 2-arm, parallel 1:1, individual randomized controlled trial conducted at the 4 health facilities from September 2020 to September 2021.

### Randomization Procedures

Eligible participants who consented to participate in the study were randomized to receive either the intervention package or standard care at the health facility in a 1:1 ratio in block sizes of 64, 96, and 128. Randomization was centralized with lists generated centrally by the FIND data manager and shared with study sites. Each site in Ghana had a separate randomization list that was handled by the field research officer and concealed from the clinical team. The randomization numbers were assigned sequentially to recruited participants, and the clinical team was made aware of the arm that a participant had been allocated to afterward to enable them to assign the participant to a prescriber.

### Patients

After providing informed consent and/or assent, participants aged 6 months to <18 years of both sexes with nonsevere acute febrile illness, defined as a temperature of >37.5°C or a history of fever within the last 7 days, who were willing to provide blood and other less invasive specimens following the protocol and available for a follow-up visit were recruited equally into the intervention or control (standard-of-care practice) arm of the study. Severely ill patients who required hospitalization were excluded. Details of the study protocol are published elsewhere [25].

### Intervention

**Figure 1.**
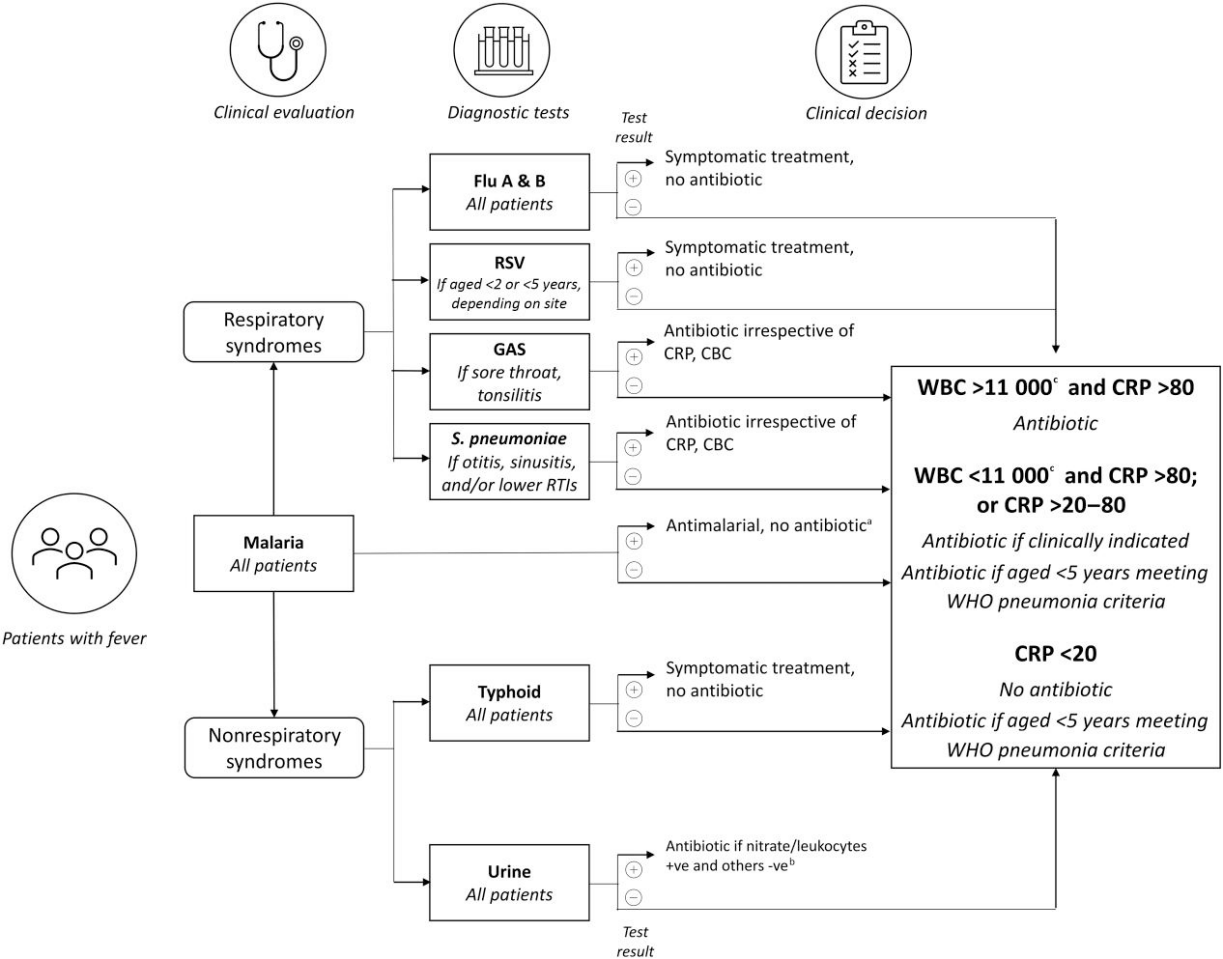
Diagnostic algorithm. ^a^Unless a concomitant bacterial pathogen identified. ^b^Start treatment followed by culture, if needed. ^c^Including neutrophils >75% if WBC >11 000 and/or neutrophils >75% if WBC <11 000. Abbreviations: CBC, complete blood count; CRP, C-reactive protein (mg/L); flu A & B, influenza A/B/A (H1B1); GAS, group A streptococci; RSV, respiratory syncytial virus; RTI, respiratory tract infection; WBC, white blood cell count (per μL); WHO, World Health Organization.

The intervention was a package of pathogen-specific and nonpathogen-specific POCTs for common causes of fever in Ghana, a clinical and diagnostic algorithm based on the results of the POCT (Figure 1), and training and communication (T&C) packages for enhancing healthcare workers’ and patients’/caregivers’ adherence to prescriptions.

**Figure 2.**
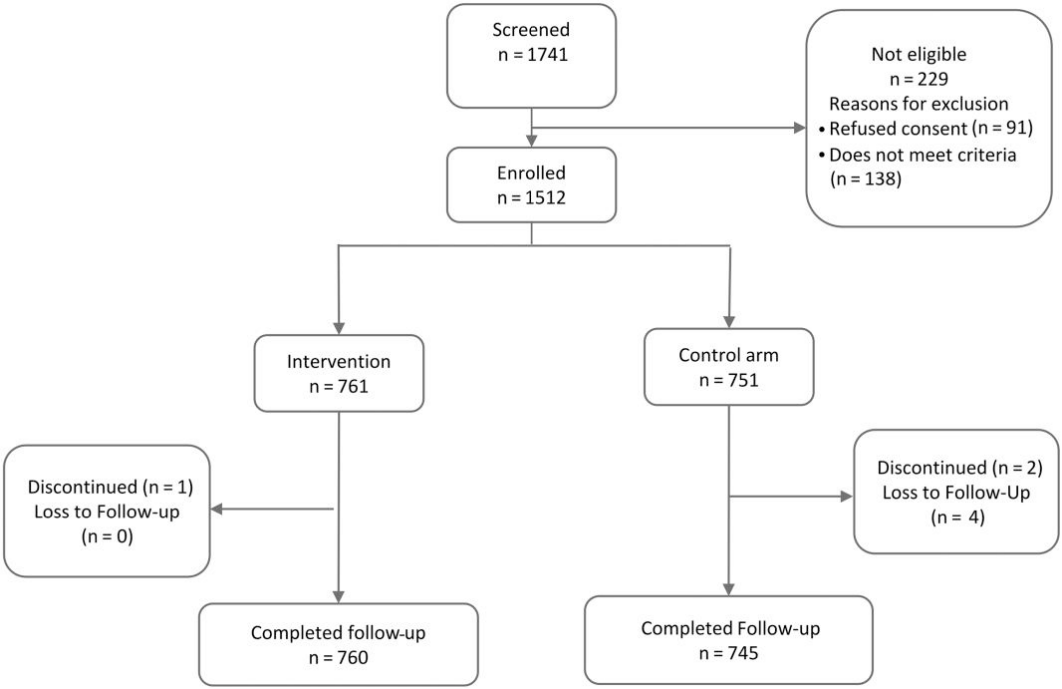
Study flow chart.

The POCTs were selected based on fever etiology in Ghana, local availability, and international approval for fever diagnosis and management. Clinical decision-making on whether or not to prescribe an antibiotic in addition to other treatments was guided by the diagnostic algorithm. When a pathogen-specific bacterial test was positive, an appropriate antibiotic was prescribed following existing guidelines. When the pathogen-specific POCT was negative, the decision to prescribe an antibiotic was based on the C-reactive protein (CRP) and white blood cell (WBC) count results. The T&C was developed based on the findings of the baseline qualitative research conducted to investigate the social, economic, and cultural factors that support or hinder patients’ adherence to antibiotic prescriptions and the communication of adherence messages from healthcare workers in the last quarter of 2019. The qualitative findings guided the development of training material for healthcare workers on adherence and how to communicate issues of adherence to participants in a patient-centered manner guided by participants’ baseline qualitative interviews. These findings have been published as a separate article in this supplement (Kukula et al).

### Objectives of the Study and End Points

The primary objectives of this study were to assess the impact of the package on clinical outcomes and antibiotic prescriptions compared with standard care for children and adolescents who present with nonsevere acute febrile illness at outpatient clinics. We calculated the proportion of patients with favorable clinical outcomes at day 7 and a prescription of any antibiotic at day 0 and compared between arms. The clinical outcome was defined as favorable if the participant was alive and well (defined as the resolution of symptoms at day 7 ±2 days with which the participant presented at day 0). The clinical outcome was unfavorable if there was persistence or worsening of symptoms present at day 0 or the development of serious adverse events.

The secondary study objectives were to assess adherence to the new diagnostic algorithm by healthcare workers, adherence to the prescriptions by patients and caregivers, and the safety of these practices. We calculated the proportion of healthcare worker actions that adhered to the algorithm, the proportion of patients who adhered to the prescription (or not) of an antibiotic, and the rates of adverse and serious adverse events per arm.

Adherence was defined as adhering to the dosage, frequency, and duration of prescribed medications including antibiotics. For participants not prescribed medications, adherence involved not buying any medications on their own, especially antibiotics, within the study period. Adherence was confirmed by pill count (≥90%) by the study pharmacist and qualitative in-depth interviews for participants who visited the clinic on day 7. The study team conducted in-depth interviews by phone for participants who could not come in for an in-person interview.

An adverse event was defined as any adverse medical occurrence (any unfavorable sign or symptom or worsening/persistent sign or symptom). A serious adverse event was defined as an adverse event that was either fatal or life-threatening, required hospitalization, or resulted in persistent or significant disability or as an important medical event that required intervention to prevent any of the aforementioned outcomes.

### Recruitment Process

Participants who presented to the study facilities were screened by study nurses in the outpatient department using the inclusion and exclusion criteria. Those who did not meet these criteria were treated as screen failures. Recruited participants were randomized into the intervention and control arms after consenting.

Participants in the intervention arm were assessed by different prescribers trained on the intervention package. A clinical history was taken, a detailed physical examination was performed, and laboratory investigations were requested. These were the POCTs that included rapid diagnostic tests (RDTs), CRP, full blood count with emphasis on the WBCs and differentials, and a urine test. The specific RDTs were influenza, respiratory syncytial virus (RSV), *Streptococcus pneumoniae*, group A streptococci, *Salmonella* Typhi, and malaria. The POC testing and reading were performed by a medical laboratory scientist and lasted about 30 minutes: 4 minutes for CRP, 1 minute for urine dipstick, about 15 minutes for other specific RDTs, and 5 minutes for WBCs. A detailed description of the POCTs and the reasons for choosing them are outlined in the introductory article to this supplement (Olliaro et al). Participants’ laboratory results were reviewed, and prescriptions including antibiotics were given based on the results, clinical algorithm, and prescriber’s experience.

The control arm followed the routine clinical flow processes of the health facilities. A clinical history was taken, and a physical examination was performed. A provisional or final diagnosis was made based on the clinical history; if necessary, laboratory testing was requested *per* national guidelines. Laboratory results were reviewed, and prescriptions were given based on the results. Different study training schedules, consulting rooms, and prescribers were used for each arm to reduce cross over contamination between arms, and the clinical team made sure there was no crossover for either participants or prescribers.

### Follow-up

Participants were followed up for 7 days for symptom resolution and adherence to the prescriptions. Follow-up visits occurred in the clinic or over the phone for those who could not come for an in-person visit. Those with persisting or worsening symptoms were reviewed, and the necessary laboratory investigations were requested for further management. Participants were interviewed on adherence to drugs given and confirmed through pill count, especially for those on antibiotics.

### Sample Size

The sample size of 2766 was calculated to obtain an estimate of the expected 30% relative reduction in antibiotic prescriptions in the intervention arm compared with the control arm. The sample size calculation was based on a 55% expected antibiotic prescription rate in the control arm, with 80% power and a significance level of 5%. Sample sizes were optimized at a margin of precision of 4% with 15% loss to follow-up rate.

### Statistical Analyses

Data were collected using offline electronic case report forms on Open Date Kit (ODK) that were later uploaded onto centralized OpenClinica software. Statistical analysis was performed using R version 4.2.1. Descriptive analysis was performed to characterize study participants, and categorical data were reported using numbers and percentages. Cross-tabulation and *χ*^2^ test analysis were calculated.

The primary outcomes were the proportion of patients with acute febrile illness with favorable clinical outcomes at day 7. The proportion of prescribed antibiotics in both arms was calculated, and the test of proportion was performed to determine any significance between the arms. This was achieved by providing point estimates of the proportion of outpatient cases of acute febrile illness with favorable outcomes (with an outcome of alive and asymptomatic) with a 95% confidence interval (CI) and comparison between arms (relative risk [RR] and absolute difference of proportions and 95% CI). Second, the point estimate of the proportion of patients with antibiotic prescriptions for acute febrile illness received on day 0 or until day 7 among all patients within the arm, comparison between the arms (RR and absolute difference of proportions and 95% CI) were estimated. Finally, we evaluated the outcomes by determining whether the patients should have received antibiotics or not based on their diagnosis.

The analysis of primary and secondary end points was described as the main analysis in a prespecified statistical analysis plan (SAP). Subgroup analysis by age, sex, and quarter of the year was also prespecified. The SAP prespecified that exploratory analyses would be conducted. No adjustment for multiple comparisons was prespecified in the SAP. Analyses by respiratory vs nonrespiratory and by malaria diagnosis were all exploratory.

Only 1512 patients were enrolled instead of the planned optimal sample size of 2766 (ie, 52%). To solve the issue of loss of power to detect the effect due to a small sample size, the precision of the analysis was adjusted from the planned 4% to 6% while maintaining the power of the study at 80% and the significance level at 5%.

### Ethics Statement

The Oxford University Clinical Trials Research Ethics Committee (Reference 52-19) and the Ghana Health Service Ethics Review Committee (Reference GHS-ERC 014/07/19) approved the study. For participants aged 6 months to 12 years, parents/guardians provided written consent, and for participants aged >12 years to <18 years, written assent was sought from them, and consent was given by their parents/guardians before engaging in the study. The study was conducted in accordance with Good Clinical Practice and the Helsinki Declaration for biomedical research on human subjects. All eligible patients gave informed consent or assent before enrollment.

## RESULTS

### Participant Demographics and Baseline Characteristics

In total, from 20 September 2020 to 31 September 2021, 1512 participants were enrolled and randomized to either the intervention (n = 761) or control (n = 751) arm from 2 main recruiting health centers as shown in Figure 2. The majority of the participants (809 of 1512, 53.5%) were male, and 1154 of 1512 (76.3%) were children aged <5 years. Participants’ baseline demographics and clinical characteristics are presented in Table 1.

**Table 1.** Patient Baseline Demographics and Characteristics

Characteristic		All Arms, All Sites	All Patients		Shai-Osudoku General Hospital Site 1		St. Andrew's Hospital Site 2	
			Intervention	Control	Intervention	Control	Intervention	Control
Total enrolled, n (%)		1512	761 (50.3)	751 (49.7)	472 (50.3)	467 (49.7)	289 (50.4)	284 (49.6)
Age, n (%), y	<5	1154 (76.3)	584 (76.7)	570 (75.9)	385 (81.6)	387 (82.9)	199 (68.9)	183 (64.4)
	5 to <10	243 (67.9)	118 (66.7)	125 (69.1)	62 (71.3)	57 (71.2)	56 (62.2)	68 (67.3)
	>10 to <18	115 (32.1)	59 (33.3)	56 (30.9)	25 (28.7)	23 (28.7)	34 (37.8)	33 (32.7)
Age, median (quarter 1, quarter 3), y		2 (1, 4)	2.00 (1, 4)	2 (1, 4)	2 (1, 4)	2 (1, 4)	3 (1, 6)	3 (1, 6)
Male sex, n (%)		809 (53.5)	399 (52.4)	410 (54.6)	250 (53.0)	257 (55.0)	149 (51.6)	153 (53.9)
Enrollment period, n (%)	July 2020–Sept. 2020	54 (3.6)	31 (4.1)	23 (3.1)	19 (4.0)	13 (2.8)	12 (4.2)	10 (3.5)
	Oct. 2020–Dec. 2020	319 (21.1)	157 (20.6)	162 (21.6)	68 (14.4)	73 (15.6)	89 (30.8)	89 (31.3)
	Jan. 2021–March 2021	286 (18.9)	144 (18.9)	142 (18.9)	64 (13.6)	64 (13.7)	80 (27.7)	78 (27.5)
	April 2021–June 2021	474 (31.3)	237 (31.1)	237 (31.6)	178 (37.7)	176 (37.7)	59 (20.4)	61 (21.5)
	July 2021–Sept. 2021	379 (25.1)	192 (25.2)	187 (24.9)	143 (30.3)	141 (30.2)	49 (17.0)	46 (16.2)
Presumptive diagnosis	Nonrespiratory, n (%)	305 (26.5)	149 (25.6)	156 (27.5)	111 (24.6)	116 (26.9)	38 (29.0)	40 (29.4)
	Respiratory, n (%)	846 (73.5)	434 (74.4)	412 (72.5)	341 (75.4)	316 (73.1)	93 (71.0)	96 (70.6)
Post-test diagnosis	Nonrespiratory, n (%)	402 (26.6)	177 (23.3)	225 (30.0)	101 (21.4)	129 (27.6)	76 (26.3)	96 (33.8)
	Respiratory, n (%)	1110 (73.4)	584 (76.7)	526 (70.0)	371 (78.6)	338 (72.4)	213 (73.7)	188 (66.2)

The median age of participants was 2 years (1–4). The most common clinical presentations were fever in 1428 of 1512 (94.4%) and/or cough in 968 of 1512 (64.0%). Typically, participants experienced symptoms for <4 days upon enrollment in the study. The mean duration (standard deviation) for fever was 2.9 (3.0) and 2.8 (1.6) days and 3.9 (3.0) and 3.6 (2.7) days for cough in the intervention and control arms, respectively. Most participants (846 of 1512, 74%) had a presumptive diagnosis of a respiratory system disease, and this was consistent at both Shai-Osudoku District Hospital and St. Andrew's Catholic Hospital. After testing, 1110 of 1512 participants (73.4%) were deemed by clinicians to have a respiratory diagnosis (Table 1, Supplementary Table 2).

### Point-of-Care Testing

In the intervention arm, all tests were performed on most participants except the *S. pneumoniae* urine antigen test, urine WBC esterase, and urine nitrites tests. All pathogen-specific tests had low positivity rates. For example, malaria antigen (Ag) tests were positive in only 84 of 738 (11.4%) participants tested, and this was consistent across arms. The most commonly identified infections were influenza A/B/A (77 positive, 10% of tests), *S. pneumoniae* (21 positive, 5.1%), group A *Streptococcus* (35 cases, 4.6%), RSV (22, 2.8%), and typhoid (12, 1.6%). In the control arm, only malaria Ag and WBC counts were performed systematically on most participants. Test results are summarized in Table 2.

**Table 2.** Point-of-Care Diagnostic Testing Results

Test	Intervention (N = 761)		Control (N = 751)	
	Tests Done, n (%)	Positive, n (%)	Tests Done, n (%)	Positive, n (%)
Malaria Pf/Pan Ag	738 (97.0)	84 (11.4)	696 (92.7)	78 (11.2)
*Salmonella* Typhi (typhoid) Immunoglobulin M	759 (99.7)	12 (1.6)	20 (2.7)	0 (0.0)
Group A *Streptococcus* Ag	758 (99.6)	35 (4.6)	8 (1.1)	1 (12.5)
Influenza A/B Ag	758 (99.6)	76 (10.0)	7 (0.9)	1 (14.3)
Respiratory syncytial virus Ag	755 (99.2)	21 (2.8)	7 (0.9)	1 (14.3)
*Streptococcus pneumoniae* urine Ag	412 (54.1)	21 (5.1)	4 (0.5)	0 (0.0)
Urine leucocyte esterase	399 (52.4)	57 (14.3)	46 (6.1)	6 (13.0)
Urine nitrites	401 (52.7)	29 (7.2)	47 (6.3)	0 (0.0)
Test	Done, n (%)	Median (Q1, Q3)	Done, n (%)	Median (Q1, Q3)
C-reactive protein	751 (98.6)	1.70 (1.00, 11.75)	22 (2.9)	1.00 (1.00, 6.30)
White blood cell count	761 (100)	8.70 (6.60, 11.60)	737 (98.1)	8.60 (6.40, 11.30)
Neutrophil count	761 (100)	45.00 (34.00, 58.00)	742 (98.8)	46.00 (34.00, 59.00)
Test	Number/Done (%)		Number/Done (%)	
CRP <20	627/751 (83.5)		19/22 (86.4)	
CRP 20 to 80	100/751 (13.3)		2/22 (9.1)	
CRP >80	24/751 (3.2)		1/22 (4.5)	
WBC <11 000	540/761 (71.0)		531/737 (72.0)	
WBC ≥11 000	221/761 (29.0)		206/737 (28.0)	
Neutrophils <75%	715/761 (94)		691/737 (93)	
Neutrophils ≥75%	46/761 (6)		51/737 (7%)	

Abbreviations: Ag, antigen; CRP, C-reactive protein (mg/L); Pf, *Plasmodium falciparum*; Q, quarter; WBC, white blood cell (per uL).

### Clinical Outcomes

By day 7, most participants in both the intervention and control arms had no fever and showed a favorable disease outcome. Only 2 of 761 (0.3%) and 4 of 751 (0.5%) participants had an unfavorable outcome in the intervention and control arms, respectively. These participants had persistent symptoms and were followed up without any additional medical treatment until the symptoms resolved. These were classified as adverse events.

### Antibiotic Prescriptions

The relative risk (RR) of an antibiotic prescription in the intervention arm was 0.89 (95% CI, .79 to 1.01) compared with the control arm. The intervention was associated with a significant reduction in antibiotic prescriptions in children aged <5 years (RR, 0.86; 95% CI, .75 to .98), patients without malaria (RR, 0.85; 95% CI, .75 to .96), patients with WBC counts <11 000/mL (RR, 0.83; 95% CI, .70 to .98), and between July 2020 and September 2020 (RR, 0.80; 95% CI, .65 to .97). Of the 2 recruiting sites, Shai-Osudoku District Hospital had a significant overall relative reduction in antibiotics prescriptions (RR, 0.81; 95% CI, .71 to .93; Table 3).

**Table 3.** Proportion of Antibiotics Prescription

Characteristic	Overall			Shai -Osudoku Hospital			St. Andrew's Hospital		
	Intervention, n/N (%) 95% CI	Control, n/N (%) 95% CI	RR [95% CI]	Intervention, n/N (%)	Control, n/N (%) 95% CI	RR [95% CI]	Intervention, n/N (%) 95% CI	Control, n/N (%) 95% CI	RR [95% CI]
All	294/760 (38.7) [35.3–42.2]	323/745 (43.4) [39.8–46.9]	.89 [.79–1.01]	202/471 (42.9) [38.5–47.4]	246/464 (53.0) [48.5–57.5]	.81 [.71–.93]	92/289 (31.8) [26.7–37.4]	77/281 (27.4) [22.5–32.9]	1.16 [.90–1.50]
Age group, y									
<5	230/583 (39.5) [35.6–43.5]	263/570 (46.1) [42.1–50.2]	.86 [.75–.98]	169/384 (44.0) [39.1–49.0]	211/387 (54.5) [49.5–59.4]	.81 [.70–.93]	61/199 (30.7) [24.7–37.4]	52/183 (28.4) [22.4–35.3]	1.08 [.79–1.47]
≥5	64/177 (36.2) [29.4–43.5]	60/175 (34.3) [27.7–41.6]	1.06 [.79–1.40]	33/87 (37.9) [28.5–48.4]	35/77 (45.5) [34.8–56.5]	.83 [.58–1.20]	31/90 (34.4) [25.4–44.7]	25/98 (25.5) [17.9–35.0]	1.35 [.87–2.10]
5 to <10	48/118 (40.7) [32.2–49.7]	46/124 (37.1) [29.1–45.9]	1.10 [.80–1.50]	26/62 (41.9) [30.5–54.3]	28/57 (49.1) [36.6–61.7]	.85 [.56–1.27]	22/56 (39.3) [27.6–52.4]	18/67 (26.9) [17.7–38.5]	1.46 [.88–2.44]
10 to <18	16/59 (27.1) [17.4–39.6]	14/51 (27.5) [17.1–40.9]	.99 [.54–1.82]	7/25 (28.0) [14.3–47.6]	7/20 (35.0) [18.1–56.7]	.80 [.34–1.90]	9/34 (26.5) [14.6–43.1]	7/31 (22.6) [11.4–39.8]	1.17 [.50–2.77]
Yearly quarter									
September 2020	6/31 (19.4) [9.2–36.3]	6/22 (27.3) [13.2–48.1]	.71 [.26–1.91]	3/19 (15.8) [5.5–37.6]	2/13 (15.4) [4.3–42.2]	1.03 [.20–5.31]	3/12 (25.0) [8.9–53.2]	4/9 (44.4) [18.9–73.3]	.56 [.17–1.91]
October 2020–December 2020	43/157 (27.4) [21.0–34.8]	48/162 (29.6) [23.1–37.1]	.92 [.65–1.31]	17/68 (25.0) [16.2–36.4]	35/73 (47.9) [36.9–59.2]	.52 [.32–.84]	26/89 (29.2) [20.8–39.4]	13/89 (14.6) [8.7–23.4]	2.00 [1.10–3.64]
January 2021–March 2021	52/143 (36.4) [28.9–44.5]	44/142 (31.0) [24.0–39.0]	1.17 [.85–1.63]	24/63 (38.1) [27.1–50.4]	21/64 (32.8) [22.6–45.0]	1.16 [.73–1.86]	28/80 (35.0) [25.5–45.9]	23/78 (29.5) [20.5–40.4]	1.19 [.75–1.87]
April 2021–June 2021	105/237 (44.39 [38.1–50.7]	119/235 (50.6) [44.3–57.0]	.88 [.72–1.06]	85/178 (47.8) [40.5–55.1]	97/176 (55.1) [47.7–62.3]	.87 [.71–1.06]	20/59 (33.9) [23.1–46.6]	22/59 (37.3) [26.1–50.0]	.91 [.56–1.48]
July 2021–September 2021	88/192 (45.8) [38.9–52.9]	106/184 (57.6) [50.4–64.5]	.80 [.65–.97]	73/143 (51.0) [42.9–59.1]	91/138 (65.9) [57.7–73.3]	.77 [.63–.95]	15/49 (30.6) [19.5–44.5]	15/46 (32.6) [20.9–47.0]	.94 [.52–1.70]
Malaria status									
RDT-positive	16/84 (19.0) [12.1–28.7]	11/75 (14.7) [8.4–24.4]	1.30 [.64–2.62]	194/423 (45.9) [41.2–50.6]	237/415 (57.1) [52.3–61.8]	.80 [.70–.92]	77/230 (33.5) [27.7–39.8]	63/201 (31.3) [25.3–38.1]	1.07 [.81–1.41]
mRDT-negative	271/653 (41.5) [37.8–45.3]	300/616 (48.7) [44.8–52.6]	.85 [.75–.96]	7/46 (15.2) [7.6–28.2]	6/31 (19.4) [9.2–36.3]	.79 [.29–2.12]	9/38 (23.7) [13.0–39.2]	5/44 (11.4) [5.0–24.0]	2.08 [.76–5.69]
Respiratory group									
Respiratory diagnosis	228/583 (39.1) [35.2–43.1]	245/526 (46.6) [42.4–50.8]	.84 [.73–.96]	41/101 (40.6) [31.5–50.3]	62/126 (49.2) [40.6–57.8]	.83 [.61–1.11]	25/76 (32.9) [23.4–44.1]	16/93 (17.2) [10.9–26.1]	1.91 [1.10–3.31]
No respiratory diagnosis	66/177 (37.3) [30.5–44.6]	78/219 (35.6) [29.6–42.2]	1.05 [.81–1.36]	161/370 (43.5) [38.6–48.6]	184/338 (54.4) [49.1–59.7]	.80 [.69–.93]	67/213 (31.5) [25.6–38.0]	61/188 (32.4) [26.2–39.4]	.97 [.73–1.29]
C-reactive protein									
<20	228/627 (36.4) [32.7–40.2]	5/19 (26.3) [11.8–48.8]	1.38 [.65–2.95]	155/387 (40.1) [35.3–45.0]	5/18 (27.8) [12.5–50.9]	1.44 [.68–3.07]	73/240 (30.4) [24.9–36.5]	0/1 (.0) [.0–79.3]	n.a.
20 to <80	43/99 (43.4) [34.1–53.3]	1/2 (50.0) [9.4–90.5]	.87 [.21–3.54]	30/61 (49.2) [37.1–61.4]	1/2 (50.0) [9.5–90.5]	.98 [.24–4.03]	13/38 (34.2) [21.2–50.1]	−/0 (n.a) [–,–]	NO DATA [n.a., n.a.]
>80	17/24 (70.8) [50.8–85.1]	1/1 (100.0) [20.6–100.0]	.71 [.55–.92]	12/17 (70.6) [46.9–86.7]	1/1 (100) [20.7–100]	.71 [.52–.96]	5/7 (71.4) [35.9–91.8]	−/0 (n.a) [–,–]	NO DATA [n.a., n.a.]
White blood cell count									
<11 000	166/540 (30.7) [27.0–34.8]	196/526 (37.3) [33.2–41.5]	.83 [.70–.98]	124/342 (36.3) [31.3–41.5]	149/324 (46.0) [40.6–51.4]	.79 [.66–.95]	42/198 (21.2) [16.1–27.4]	47/202 (23.3) [18.0–29.6]	.91 [.63–1.32]
>11 000	128/220 (58.2) [51.6–64.5]	125/206 (60.7) [53.9–67.1]	.96 [.82–1.12]	78/129 (60.5) [51.8–68.5]	96/135 (71.1) [63.0–78.1]	.85 [.71–1.01]	50/91 (54.9) [44.7–64.8]	29/71 (40.8) [30.2–52.5]	1.35 [.96–1.88]
Neutrophils									
<75%	268/714 (37.5) [34.1–41.1]	292/687 (42.5) [38.9–46.2]	.88 [.78–1.00]	185/443 (41.8) [37.3–46.4]	224/433 (51.7) [47.0–56.4]	.81 [.70–.93]	83/271 (30.6) [25.4–36.4]	68/254 (26.8) [21.7–32.5]	1.14 [.87–1.50]
>75%	26/46 (56.5) [42.2–69.8]	29/50 (58.0) [44.2–70.6]	.98 [.69–1.38]	17/28 (60.7) [42.4–76.4]	21/29 (72.4) [54.3–85.3]	.84 [.58–1.22]	9/18 (50.0) [29.0–71.0]	8/21 (38.1) [20.8–59.1]	1.31 [.64–2.68]

Abbreviations: CI, confidence interval; n.a., not applicable; RDT, rapid diagnostic test; RR, relative risk.

There was a significant reduction in prescriptions of antibiotics for patients who had a confirmed (post-test) diagnosis categorized as a respiratory disease (RR, 0.84; 95% CI, .73 to .96). This was not the case for those diagnosed with nonrespiratory diseases, the majority of whom had a diagnosis other than malaria. However, even among these patients with a nonrespiratory diagnosis, the intervention arm had significantly reduced antibiotic prescriptions among those not tested for malaria (RR, 0.57; 95% CI, .42 to .79; Supplementary Table 1).

### Antibiotic Prescription Adherence

Adherence to antibiotic prescriptions by patients during the study was high, with most patients completing their antibiotic prescriptions in both the intervention and control arms (117 of 131, 89.3% vs 160 of 171, 93.6%, respectively; *P* = .263) as assessed by pill counts (Table 5).

Adherence to the prescription as measured by qualitative methods was high with no difference between arms, 277 of 293 (94.5%) in the intervention arm and 308 of 321 (96.0%) in the control arm (diff = −1.4%; 95% CI, −5.1 to 2.3). Adherence to prescriptions was high in both arms, irrespective of the clinical outcome. No significant difference in adherence was seen between the arms by combining pill counts and qualitative interviews. Adherence to prescription was also high among patients without an antibiotic prescription, and there was no difference between arms.

### Healthcare Worker Adherence to the Algorithm

Adherence to the clinical algorithm by prescribers showed that there were 52 participants who were not supposed to receive antibiotics but were prescribed antibiotics, and a similar number were prescribed antibiotics even though the algorithm suggested otherwise. The number of patients who were prescribed antibiotics was proportionally higher at Shai-Osudoku District Hospital (41 of 120, 34.2%) compared with St. Andrew's Hospital (11 of 78, 14.1%). There was a comparable proportion across the 2 sites for those who were supposed to receive an antibiotic but were not prescribed (28 of 83, 33.7% vs 24 of 65, 36.9%; Table 4).

**Table 4.** Adherence to Algorithm by Healthcare Workers

Algorithm (“As Described”) Implication	All Sites						Shai Osudoku District Hospital						St. Andrew's Hospital			
	Antibiotic Prescription						Antibiotic Prescription						Antibiotic Prescription			
	“Missing”		No		Yes		“Missing”		No		Yes		No		Yes	
	n	%	n	%	n	%	n	%	n	%	n	%	n	%	n	%
Don´t prescribe antibiotic	0	0	146	19.2	52	6.8	0	0	79	16.7	41	8.7	67	23.2	11	3.8
Prescribe antibiotic, if indicated	0	0	268	35.2	147	19.3	0	0	162	34.3	107	22.7	106	36.7	40	13.8
Prescribe antibiotic	1	0.1	52	6.8	95	12.5	1	0.2	28	5.9	54	11.4	24	8.3	41	14.2
Total	1	0.1	466	61.2	294	38.6	1	0.2	269	57.0	202	42.8	197	68.2	92	31.8

**Table 5.** Adherence to Antibiotic Prescription at Day 0 in Patients Managed in Intervention and Control Arms

Characteristic	Overall, n/N (%)		Intervention Arm, n/N (%)		Control Arm, n/N (%)		Risk Difference, % [95% Confidence Interval]	*P* Value
	Antibiotic	No Antibiotic	Antibiotic	No Antibiotic	Antibiotic	No Antibiotic		
Antibiotic prescribed	614 (40.8)	890 (59.2)	293 (38.6)	467 (61.4)	321 (43.1)	423 (56.9)	−4.6% [−9.7–.5]	.079
Adherence A (by qualitative interview)	585/614 (95.3)	…	277/293 (94.5)	…	308/321 (96.0)	…	−1.4% [−5.1–2.3]	.527
Adherence B (by pill counts)	277/302 (91.7)	…	117/131 (89.3)	…	160/171 (93.6)	…	−4.3% [−11.4–2.9]	.263
Adherence C (both interview and pill count)	275/316 (87.0)	…	116/140 (82.9)	…	159/176 (90.3)	…	−7.5% [−15.7–.8]	.072
Adherence D (adherence to a no- antibiotic prescription)	…	887/890 (99.7)	…	466/467 (99.8)	…	421/423 (99.5)	.3% [−.7–1.3]	.932
Adherence A + D	1472/1504 (97.9)		743/760 (97.8)		729/744 (98.0)		−.2% [−1.8–1.4]	.906
Adherence B + D	1164/1192 (97.7)		583/598 (97.5)		581/594 (97.8)		−.3% [−2.2–1.6]	.862
Adherence C + D	1162/1206 (96.4)		582/607 (95.9)		580/599 (96.8)		−.9% [−3.2, 1.3]	.470

A*: Accounting for the following: “Completed treatment as reported through qualitative in-depth interviews or directly to the healthcare worker.”

B*: Accounting for the following: “Pill count criterion (≥90%).”

C*: Accounting for the following: “Completed treatment” and “Pill count criterion (≥90%).”

D**: Accounting for the following: “No other antibiotic (in participants without prescription on day 0).”

* “Patient bought antibiotic” and “Other antibiotic” were common criteria for A, B, and C.

** The nonadherent cases directly reflect cases being referred to as “antibiotic taken.”

### Withdrawal

Four participants (2 from each arm) were withdrawn on day 1 for in-patient care and treated for severe adverse events; 3 had high malaria parasite counts, and the fourth had tonsillitis with an elevated WBC count of 27.7 × 10^9^/L with 62% neutrophils. All participants were discharged after 48 hours of parenteral treatment and had favorable clinical outcomes when followed up on day 7.

## DISCUSSION

We aimed to compare the impact of point-of-care rapid diagnostic tests in a diagnostic algorithm on antibiotic prescriptions for acute febrile illness as well as clinical outcomes and adherence of patients and caregivers. The majority of enrolled patients had a favorable outcome in terms of resolution of fever and other clinical symptoms in both arms, irrespective of the treatment received.

Overall, there was no statistically significant reduction in antibiotic prescriptions in the intervention arm. However, there were significant reductions when antibiotic prescriptions were stratified by age and disease: in children aged <5 years and in patients without malaria (RDT-negative) and with respiratory symptoms and diagnosed as having respiratory diseases. This is in line with the results of the same study conducted in Burkina Faso and Uganda during the same period (September 2020 to September 2021).

The difference in antibiotic prescriptions across the 2 arms in our study, even though smaller and not close to the anticipated 30% reduction, reveals the role of POCTs, which are effective in primary care settings in the fight against inappropriate antibiotic prescription and antibiotic resistance [1, 2]. This is significant in the fight against antimicrobial resistance because a single resistant bacteria in any environment can erode the gains made to date. The level of antibiotic prescription in both arms was still higher than the WHO-recommended optimum level of less than 30% of encounters among primary healthcare providers [26]. One effective and efficient way to achieve this is to provide affordable, rapid POCTs that can be easily integrated into the primary healthcare setting to guide prescribers in making evidence-based decisions [27].

The proportion of antibiotics prescribed in the intervention arm was lower compared with similar studies that examined acute febrile illness in Ghana at 70.1% [10]. A significant difference is that most participants (76.3%) in our study were aged <5 years compared with the Ghana study. With age documented as a significant factor in the choice of antibiotic prescription, this discordance is avoidable if we follow guidelines such as the WHO AWaRe (access, watch, reserve) antibiotic handbook and/or local guidelines [23, 28]. Other studies have shown a similar higher proportion of antibiotic prescriptions in children, even with different age distributions compared with our study. This reflects the burden and the possibility of overprescription among the pediatric population, which is commonly affected by acute febrile illness [6,8].

A recent point prevalence survey conducted in a tertiary health facility in Ghana showed the proportion of antibiotic prescriptions to be 51.4% [5]. This could be due to the different levels of care offered in a tertiary facility where complicated cases are managed compared with the primary care offered in our study areas. Nevertheless, there is a need to further explore the reasons and factors behind these prescription levels to understand and address them. Other studies have recorded comparable proportions of 36% antibiotic prescribing from electronic medical records of patients in a district hospital in Ghana [8].

A high level of adherence to prescriptions was observed in both arms. This could not be attributed to the impact of the behavioral change communication that was administered only to the intervention arm. Adherence at the participant level is usually influenced by different determinants, and it will be interesting to conduct future studies in order to determine the reasons behind this high level in the context of the study area. It is important not to discount the possible effect of residual contamination among participants through communication at the household level and among prescribers through work interactions despite the steps taken to minimize it.

Limitations of our study include the significantly low levels of confirmed respiratory infections by the rapid diagnostic tests. This could be explained by the period in which the study was undertaken. The study was conducted during the coronavirus disease 2019 pandemic just after the first major wave and throughout the second wave [29]. Schools that normally serve as fertile ground for respiratory tract infection transmission were closed with prospective study patients on lockdown break. This is reflected in the small number of patients who presented at health facilities. Nevertheless, viral infections (flu and RSV) were the most diagnosed and confirmed in other studies, especially in the age group of the study participants. Given that the study was not set up to examine the presence of severe acute respiratory syndrome coronavirus 2, we are only able to report our results for infections that were tested [30, 31]. Another limitation is the effect of potential contamination of the control arm regarding the level of antibiotic prescription and the difficulty in measuring or underestimating the effect of this on the interventional package.

## CONCLUSIONS

At the primary healthcare level, we show that the intervention package of POCTs, diagnostic algorithms, and behavioral change communication for healthcare workers can reduce antibiotic prescriptions. Children aged <5 years, and patients who present with respiratory febrile illnesses confirmed to be non-malarial, were the patient groups most impacted.

There is a need for policymakers to incorporate simple POCTs into primary healthcare systems to help prescribers make appropriate decisions regarding antibiotic usage in low- and middle-income countries.

## Supplementary Data

Supplementary materials are available at *Clinical Infectious Diseases* online. Consisting of data provided by the authors to benefit the reader, the posted materials are not copyedited and are the sole responsibility of the authors, so questions or comments should be addressed to the corresponding author.

## Supplementary Material

ciad328_Supplementary_Data

## Notes

***Author contributions*.** A. A., V. K., J. W., R. B., P. O., P. H., S. D., C. E. M., and O. S. were involved in the conception and design of the study. A. A., V. K., C. T. N., S. O., E. A., G. O., M. M. M., P. H., O. S., E. A., J. N., and R. B. were involved in the acquisition of data. C. N., E. A., G. O., and M. M. were involved in data analysis. A. A., J. W., and R. B. wrote the first draft. All authors were involved in data interpretation and refining the manuscript, and all authors reviewed and approved the final version of the manuscript.

***Acknowledgments*.** The authors are grateful to the participating patients and caregivers and to the staff of the health centers that participated in the study.

***Financial support*.** This study was funded by the Foreign Commonwealth and Development Office (FCDO), United Kingdom, Swiss Agency for Development and Cooperation, and the German Federal Ministry of Economic Cooperation and Development (BMZ).

***Supplement sponsorship*.** This article appears as part of the supplement “Using Diagnostic Tools to Support Antimicrobial Stewardship and Improve Outcome in Resource-Limited Contexts,” sponsored by FIND.
