# Supplementary material for: Impact of Point-of-Care Rapid Diagnostic Tests on Antibiotic Prescription Among Patients Aged <18 Years in Primary Healthcare Settings in 2 Peri-Urban Districts in Ghana: Randomized Controlled Trial Results
Source: Clin Infect Dis. 2023 Jul 25;77(Suppl 2):S145–55. doi: 10.1093/cid/ciad328 (PMC10368405; doi:10.1093/cid/ciad328)
Supplement: ciad328_Supplementary_Data [file ciad328_supplementary_data.docx]

## Supplementary material

ST1: Antibiotic prescriptions in confirmed diagnoses of a randomized controlled trial looking at the impact of point-of-care rapid diagnostic test of antibiotic prescription among patients under 18 years in primary health care settings in two peri-urban districts in Ghana

| **Diagnosis in non-respiratory group** | **Intervention arm** | **Control arm** | **Relative Risk [95%CI]** |
| --- | --- | --- | --- |
| Malaria | 20.0%  [ 13.2% to 29.1%] (n=19 of N=95) | 14.3%  [ 8.2% to 23.8%] (n=11 of N=77) | 1.40  [ 0.71 to 2.76] |
| No-malaria | 46.0%  [ 41.8% to 50.3%] (n=238 of N=517) | 48.4%  [ 43.7% to 53.1%] (n=211 of N=436) | 0.95  [ 0.83 to 1.09] |
| No confirmed diagnosis | 25.0%  [ 18.7% to 32.6%] (n=37 of N=148) | 43.5%  [ 37.3% to 50.0%] (n=101 of N=232) | 0.57  [ 0.42 to 0.79] |

CI: confidence interval.

ST2: Patient baseline characteristics of a randomized controlled trial looking at the impact of point-of-care rapid diagnostic test of antibiotic prescription among patients under 18 years in primary health care settings in two peri-urban districts in Ghana

|  | **Intervention**  **(N=761)** | **Control**  **(N=745)** |
| --- | --- | --- |
| **Age (years)**, mean (SD) | 3 (3.4) | 3 (3.3) |
| **Weight (kg)**, mean (SD) | 15.6 (10.2) | 15.6 (10.0) |
| **Height (cm)**, mean (SD) | 95.4 (23.2) | 95.7 (22.4) |
| **Male**, n (%) | 398 (52) | 407 (55) |
| **Occupation**, n (%) |  |  |
| Student | 348 (46) | 315 (42) |
| Pre-school child | 412 (54) | 439 (58) |
| **Educational Level** |  |  |
| Early childhood education | 354 (47) | 365 (49) |
| Lower secondary education | 17 (2) | 19 (3) |
| Primary education | 128 (17) | 118 (16) |
| None | 264 (35) | 243 (33) |
| **Presumptive diagnose (before tests)** |  |  |
| No diagnosis term | 178 (23%) | 183 (24%) |
| Non-respiratory diagnoses | 149 (20%) | 156 (21%) |
| Respiratory diagnoses | 434 (57%) | 412 (55%) |
| **Post-test diagnosis (confirmed diagnosis)** |  |  |
| Respiratory diagnosis | 584 (77%) | 526 (70%) |
| Non-respiratory diagnosis | 177 (23%) | 225 (30%) |
| **Patient symptoms**,* n (%) / mean duration in days (SD) |  |  |
| Fever | 716 (94) / 2.9 (3.0) | 712 (96) / 2.8 (1.6) |
| Cough | 497 (65) / 3.9 (3.0) | 471 (63) / 3.6 (2.7) |
| Sneezing | 374 (49) / 3.7 (2.7) | 340 (46) / 3.4 (2.2) |
| Vomiting | 184 (24) / 1.9 (1.7) | 169 (23) / 1.9 (1.5) |
| Headache | 151 (20) / 2.8 (1.9) | 155 (21) / 2.7 (1.9) |
| Abdominal pain | 109 (14) / 2.6 (1.7) | 117 (16) / 2.5 (2.3) |
| Diarrhoea | 104 (14) / 3.1 (2.6) | 108 (15) / 3.0 (1.9) |
| Sore throat | 42 (6) / 3.1 (1.8) | 50 (7) / 2.6 (1.7) |
| Rash | 40 (5) / 8.0 (8.5) | 48 (6) / 9.0 (13.8) |
| **Vital signs**, mean (SD) |  |  |
| Heart rate (bpm) | 121.1 (21.3) | 122.7 (19.0) |
| Blood pressure (systolic, mm Hg) | 114.5 (12.7) | 109.0 (8.9) |
| Blood pressure (diastolic mm Hg) | 69.7 (9.1) | 68.7 (8.3) |
| Temperature, (^0^C) | 36.9 (0.7) | 36.9 (0.7) |
| **Patient examination findings**, † n (%) |  |  |
| *General appearance* |  |  |
| Acutely ill | 5 (1) | 6 (1) |
| Chronically ill | 0 | 1 (<1) |
| Moderately ill | 324 (43) | 339 (46) |
| Not ill | 431 (57) | 339 (54) |
|  |  |  |
| *Ear, nose and throat (positive)* | n=271 | n=240 |
| Pharyngeal erythema | 94 (35) | 67 (28) |
| Pharyngeal enlargement | 80 (30) | 76 (32) |
| Hyperemic tympanic membrane | 58 (21) | 60 (25) |
| Other | 39 (14) | 37 (15) |
| *Skin* | n=49 | n=59 |
| Papular rash | 16 (33) | 16 (27) |
| Pallor | 12 (24) | 15 (25) |
| Nodular rash | 4 (8) | 3 (5) |
| Impetigo | 5 (10) | 8 (14) |
| Other | 17 (35) | 25 (42) |

*Occurring in >5% of patients overall (n=76); ^†^For testing groups (e.g., ‘skin’) where positive findings were found in >5% of patients overall.

SD: standard deviation.
